# Fecal, duodenal, and tumor microbiota composition of esophageal carcinoma patients, a longitudinal prospective cohort

**DOI:** 10.1093/jnci/djae153

**Published:** 2024-06-26

**Authors:** Tom van den Ende, Nicolien C de Clercq, Mark Davids, Ruben Goedegebuure, Benthe H Doeve, Gati Ebrahimi, Jeroen Buijsen, Ronald Hoekstra, Nadia Haj Mohammad, Maarten F Bijlsma, Max Nieuwdorp, Hanneke W M van Laarhoven

**Affiliations:** Department of Medical Oncology, Amsterdam University Medical Center, University of Amsterdam, Amsterdam, the Netherlands; Cancer Center Amsterdam, Imaging and Biomarkers, Amsterdam, the Netherlands; Department of Medical Oncology, Amsterdam University Medical Center, University of Amsterdam, Amsterdam, the Netherlands; Cancer Center Amsterdam, Imaging and Biomarkers, Amsterdam, the Netherlands; Department of Internal and Vascular Medicine, Amsterdam University Medical Center, University of Amsterdam, Amsterdam, the Netherlands; Department of Internal and Vascular Medicine, Amsterdam University Medical Center, University of Amsterdam, Amsterdam, the Netherlands; Netherlands Cancer Institute, Gastrointestinal Oncology, Amsterdam, the Netherlands; Department of Medical Oncology, Amsterdam University Medical Center, University of Amsterdam, Amsterdam, the Netherlands; Cancer Center Amsterdam, Imaging and Biomarkers, Amsterdam, the Netherlands; Oncode Institute, Utrecht, the Netherlands; Center for Experimental and Molecular Medicine, Amsterdam University Medical Center, University of Amsterdam, Laboratory for Experimental Oncology and Radiobiology, Amsterdam, the Netherlands; Department of Radiotherapy, Instituut Verbeeten, Tilburg, the Netherlands; Department of Radiation Oncology (MAASTRO), School for Oncology and Developmental Biology (GROW), Maastricht University Medical Center, Maastricht, the Netherlands; Department of Medical Oncology, Ziekenhuisgroep Twente, Hengelo, the Netherlands; Department of Medical Oncology, University Medical Center Utrecht, Utrecht University, Utrecht, the Netherlands; Oncode Institute, Utrecht, the Netherlands; Center for Experimental and Molecular Medicine, Amsterdam University Medical Center, University of Amsterdam, Laboratory for Experimental Oncology and Radiobiology, Amsterdam, the Netherlands; Cancer Center Amsterdam, Cancer Biology and Immunology, Amsterdam, the Netherlands; Department of Internal and Vascular Medicine, Amsterdam University Medical Center, University of Amsterdam, Amsterdam, the Netherlands; Department of Medical Oncology, Amsterdam University Medical Center, University of Amsterdam, Amsterdam, the Netherlands; Cancer Center Amsterdam, Imaging and Biomarkers, Amsterdam, the Netherlands

## Abstract

**Background:**

The microbiome has been associated with chemotherapy and immune checkpoint inhibitor efficacy. How this pertains to resectable esophageal carcinoma is unknown. Our aim was to identify microbial signatures in resectable esophageal carcinoma associated with response to neoadjuvant chemoradiotherapy with or without an immune checkpoint inhibitor.

**Methods:**

From 2 prospectively collected esophageal carcinoma cohorts (n = 172 in total) treated with neoadjuvant chemoradiotherapy alone (n = 132) or a combination of neoadjuvant chemoradiotherapy and an immune checkpoint inhibitor (n = 40), fecal samples were available at baseline, during treatment, and presurgery. Additionally, in the immune checkpoint inhibitor–treated patients, tumor and duodenal snap frozen biopsies were collected over time. Fecal, tumor, and duodenal DNA were extracted for 16S ribosomal RNA sequencing. Associations were investigated between microbiome composition pathological complete response and progression-free survival (PFS).

**Results:**

There was a statistically significant shift in the microbiota profile of the fecal, tumor, and duodenal microbiota over time. In the total cohort, patients with a pathological complete response had a stable fecal alpha diversity, while the diversity of poor responders decreased during treatment (*P* = .036). Presurgery, lower alpha diversity (<4.12) was related to worse PFS (log-rank *P* = .025). Baseline tumor biopsies of patients with short PFS had more *Fusobacterium*. A low baseline duodenal alpha diversity (<3.96) was associated with worse PFS (log-rank *P* = .012).

**Conclusions:**

Lower intestinal alpha diversity was associated with worse response and survival of esophageal carcinoma patients. In tumor biopsies, *Fusobacterium* was more abundant in patients with poor PFS. After further mechanistic validation, these findings may aid in response prediction and the design of novel microbiome modulating treatments for esophageal carcinoma patients.

Resectable esophageal cancer can be treated with neoadjuvant chemoradiotherapy or perioperative chemotherapy (1). The standard neoadjuvant chemoradiotherapy regimen in several countries consists of the 5-week CROSS regimen with weekly carboplatin and paclitaxel for both histological subtypes (2). Recently, the CheckMate 577 trial established the anti–programmed cell death 1 inhibitor nivolumab as a new adjuvant treatment option after CROSS for incomplete responders (3). Despite these advances in esophageal carcinoma treatment, the long-term outcome of these patients is still poor with recurrence rates up to 50% within 5 years (4). To further improve treatment response of established protocols, additional therapeutic approaches are needed.

The human microbiome could serve this purpose as it has been linked to the efficacy of anticancer therapy including immune checkpoint inhibitors, chemotherapy, and radiotherapy (5). Several mechanisms of action exist through which the gut and intratumoral microbiome can affect treatment efficacy by modulating immunity, excretion of metabolites or microbe-associated molecular patterns, and pharmacodynamic or genetic mechanisms (6). Multiple bacterial species from the gut have been identified as modulators of immune checkpoint inhibitor response based on clinical observations and in vivo research (7-10). The role of the microbiome also extends to the efficacy of chemotherapy with fecal and intratumoral bacterial species linked to therapy response (11-14). Moreover, in a recently published randomized intervention study from our research group, an allogenic fecal microbiota transplant of obese donors improved chemotherapy response in cachectic metastatic gastroesophageal cancer patients (15). Preliminary evidence from in vivo and small cohort studies also suggests an impact of the fecal microbiota on radiotherapy and chemoradiotherapy response (16-18). Taken together, these results provide hints towards a role for the host and tumor microbiome in response to chemoradiotherapy and immune checkpoint inhibitor therapy.

Orodigestive dysbiosis, intratumoral microbes such as *Fusobacterium nucleatum,* and gut microbes have already been associated with the presence of esophageal cancer, its disease stage, and survival (19-23). The local tumor biome interacts directly with cancer cells, but also more indirect pathways exist between the fecal microbiome and tumor progression or treatment response, such as microbiota-derived metabolites (24). These metabolites are involved in oncogenesis in many ways, such as interaction with immune responses and regulating signaling pathways of cancer-related genes (25). Previous observational studies were primarily performed in patients with esophageal squamous cell carcinoma and lack longitudinal sampling during neoadjuvant therapy. To further shed light on the relationship between the microbiome and treatment response, we conducted a prospective longitudinal sampling study in locally advanced, nonmetastatic esophageal carcinoma patients treated with neoadjuvant chemoradiotherapy. The aim of our study was to investigate the relationship between the microbiome and response to therapy. Additionally, we investigated the tumor and duodenal microbiota in patients treated with neoadjuvant checkpoint blockade.

## Patients and methods

### Study cohorts

In this study, we included 172 patients with esophageal carcinoma from 2 prospective cohorts, IMPAC and PERFECT, treated with neoadjuvant chemoradiotherapy. The IMPAC fecal collection cohort consisted of 132 patients with resectable stage II-III esophageal and gastroesophageal junction tumors scheduled for treatment with neoadjuvant chemoradiotherapy according to the CROSS regimen (2). Patients were included from both histological subtypes: esophageal adenocarcinoma and esophageal squamous cell carcinoma. We excluded patients with an active malignancy affecting the prognosis of esophageal cancer, as well as those with active infections or prior cytotoxic therapy. Patients were recruited to participate in the IMPAC study from the Amsterdam University Medical Center location Meibergdreef and De Boelelaan, Hospital Group Twente, Maastro Clinic, Radiotherapy Institute Verbeeten and University Medical Center Utrecht. The immune checkpoint inhibitor–treated cohort (PERFECT) consisted of 40 patients with stage II-III resectable esophageal adenocarcinoma or gastroesophageal junction tumors treated with neoadjuvant chemoradiotherapy combined with antiprogrammed cell death-1 ligand blockade in a phase II clinical trial (NCT03087864) (26). Inclusion and exclusion criteria have previously been described (26). Patients were included in the Amsterdam University Medical Center and University Medical Center Utrecht. The medical ethics committee of the Amsterdam University Medical Center location Meibergdreef evaluated and approved the PERFECT and IMPAC studies. All patients provided written, informed consent for study participation. This study was conducted in accordance with the Declaration of Helsinki and the international standards of good clinical practice.

### Sample collection

**Figure 1.**
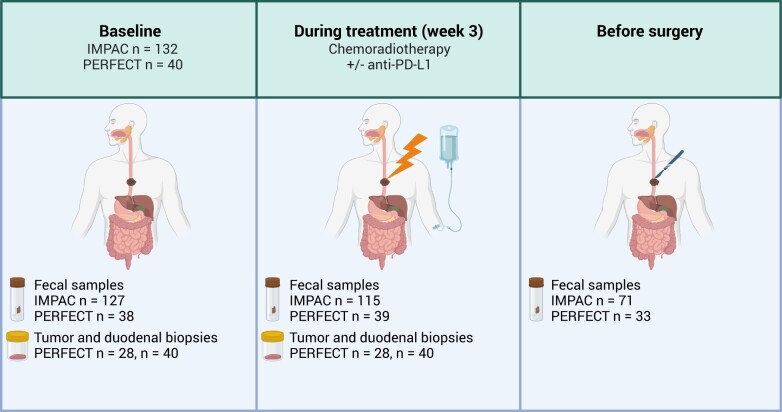
Treatment and sampling timepoints for IMPAC and PERFECT. Fecal samples were collected in both cohorts, while tumor and duodenal samples were only collected in the immunotherapy-treated patients from PERFECT. PD-L1 = programmed cell death 1 ligand.

Fecal samples for gut microbiome characterization were collected in feces tubes (Sarstedt Inc, Nümbrecht, Germany) at 3 timepoints: baseline, during treatment (week 3 of neoadjuvant chemoradiotherapy), and before surgery (postneoadjuvant therapy). In the IMPAC cohort, baseline fecal samples were available from 127 patients, during treatment from 115 patients, and before surgery from 71 patients. No baseline samples were available from 7 patients. In the immune checkpoint inhibitor cohort baseline, fecal samples were available from 38 patients, during treatment from 39 patients, and before surgery from 33 patients. From the immune checkpoint inhibitor–treated PERFECT cohort, we also collected gastroscopy-derived snap frozen esophageal adenocarcinoma tumor and duodenal biopsies at baseline and during treatment for microbial analysis from 28 and 40 patients, respectively (see Figure 1).

### Clinical data and outcome measures

Clinical data were collected from patient records including age, sex, body mass index, smoking status, alcohol use, prior cholecystectomy, diabetes, use of proton pomp inhibitors, antibiotics, antifungal agents, diabetes, and nasogastric tube feeding (27). Additionally, we examined tumor variables including T stage and N stage, histology subtype, grade, and tumor length. Outcome measures were pathological complete response to neoadjuvant chemoradiotherapy and progression-free survival (PFS). Response was measured according to the ypTNM stage. Assessment of response was done by comparing patients with a pathological complete response (ypT0N0) to patients with residual cancer (ypT+, ypN+) or progression before surgery. PFS was defined as disease progression, recurrence or death measured from the start of treatment. Patients were censored at the end of follow-up. Data cutoff for survival was June 21, 2022. To dichotomize PFS for the microbiome analyses, 2 groups were compared: short PFS (disease progression, recurrence, or death within 43 months after start of treatment) vs long PFS (no event within follow-up time).

### Microbiome analyses

As previously described by de Groot et al. (28), DNA was extracted from fecal material using a repeated bead beating protocol (29). DNA was purified using Maxwell RSC Whole Blood DNA Kit. 16S ribosomal RNA gene amplicons were generated using a single-step polymerase chain reaction protocol targeting the V3-V4 region (30). Polymerase chain reaction products were purified using Ampure XP beads, and purified products were equimolar pooled. The libraries were sequenced using a MiSeq platform using V3 chemistry with 2 x 251 cycles. Forward and reverse reads were truncated to 240 and 210 bases, respectively, and merged using USEARCH (31). Merged reads that did not pass the Illumina chastity filter had an expected error rate higher than two or were shorter than 380 bases. Amplicon sequence variants were inferred for each sample individually from sequences with a minimum abundance of 4 reads (31). Unfiltered reads were then mapped against the joint amplicon sequence variant set to determine per-sample amplicon sequence variant abundances. Taxonomy was assigned using the RDP classifier and SILVA 16S ribosomal database V132 (32,33). Fecal duodenal and tumor samples were rarefied to 5000 counts. Additionally, for the tumor and duodenal samples, amplicons with a size less than 300 base pairs were removed to filter out artificial and mitochondrial reads. From the fecal samples, 3 (n = 2 baseline, n = 1 during treatment) had to be removed because there were not enough counts. From the tumor biopsies, 9 samples (n = 5 baseline, n = 4 during treatment) had to be removed, and from the duodenal biopsies 21 samples (n = 7 baseline, n = 14 during treatment) because they did not pass the filter criteria.

### Statistical analysis

Effects in time were assessed using multilevel principal components analysis (PCA), on clr-transformed data, using the mixOmics package (34). Statistical significance was tested using a permutation multivariate analysis of variance (MANOVA) on the first 10 components. Beta diversity (Bray–Curtis dissimilarity) was tested by permutational multivariate analysis of variance from Vegan to test differences in microbiota composition (35). Differences in alpha diversity were measured by the Shannon index. Beta diversity measures the similarity or dissimilarity in microbiome composition between 2 groups of samples, and alpha diversity assesses community heterogeneity within a single sample. Differential abundance of taxa was tested using DESeq2. Statistical analyses and visualizations were performed in R (v. 3.6.2) using the ggplot2 package. Nonparametric tests were used to assess differences between timepoints and patient outcome (Wilcoxon signed-rank test). The Benjamini–Hochberg procedure was used to correct *P* values for multiple comparisons. Cutoff Finder was used to divide patients into high- and low-risk groups by the log-rank test based on the Shannon index (36). Analyses for the fecal microbiota were performed on all participants (n = 172) of the 2 cohorts unless otherwise stated. An α less than 0.05 was regarded as statistically significant. All statistical tests were conducted 2-sided.

## Results

### Effect of neoadjuvant therapy on gastrointestinal microbiome composition

**Figure 2.**
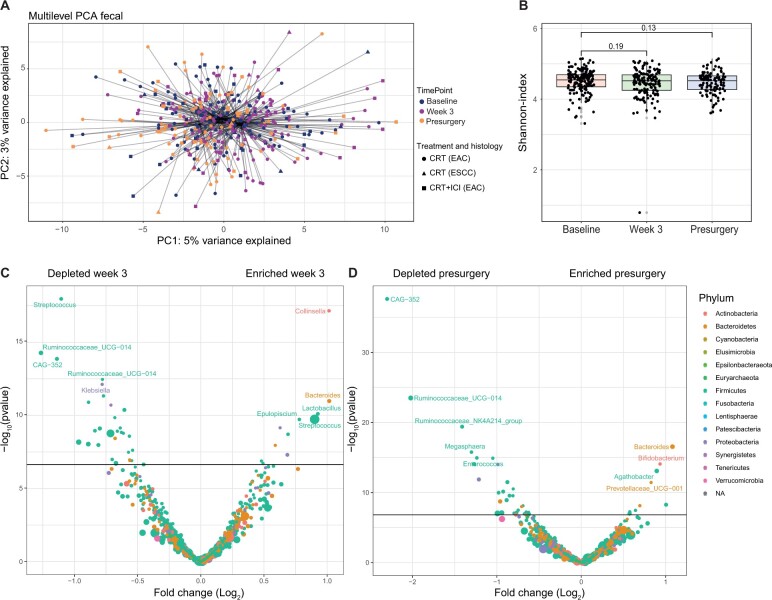
Fecal microbiota dynamics over time. **A)** Multilevel principal component analysis of fecal microbiota samples over time. The colors are indicative of the 3 timepoints, while symbols indicate treatment received and tumor histology. **B)** Shannon index over time for the fecal microbiota samples. **C)** Volcano plot of fecal bacteria more or less abundant in week 3 compared with baseline. **D)** Volcano plot of fecal bacteria more or less abundant presurgery compared with baseline. CRT = chemoradiotherapy; EAC = esophageal adenocarcinoma; ESCC = esophageal squamous cell carcinoma; ICI = immune checkpoint inhibitor; NA = not applicable; PC1 = first principal component; PC2 = second principal component; PCA = principal components analysis.

The fecal samples from the IMPAC cohort (n = 132 patients) were prospectively collected between September 2018 and March 2022 (Figure 1). The total number of esophageal adenocarcinoma patients was 110, and the number of esophageal squamous cell carcinoma patients was 22. In the PERFECT trial (n = 40 patients, all with esophageal adenocarcinoma), fecal, tumor, and duodenal samples were prospectively collected between July 2017 and March 2019 (Figure 1). Baseline characteristics are shown in Table 1. First, we assessed the difference in fecal microbiota composition in serial samples during and after treatment. The composition on genus level for each timepoint is given in Supplementary Figure 1 (available online). The between-sample similarity or dissimilarity was investigated by comparing the 3 timepoints on multilevel PCA (Figure 2, A). A statistically significant shift in composition was observed (MANOVA, *P* < .001). Neoadjuvant treatment explained 5% of the variance in fecal microbiota composition. There was no separate clustering of samples according to treatment regimen or histology. Next, we investigated if alpha diversity (Shannon index) changed over time. There was no statistically significant difference between baseline and week 3 (*P* = .19) or baseline and presurgery (*P* = .13; Figure 2, B). To compare differential abundance of individual bacteria, we compared the baseline samples with the on-treatment week 3 or presurgery samples (Figure 2, C and D). In week 3, there was an increase in *Collinsella*, *Bacteroides*, and *Lactobacillus*, whereas there was a decrease in several firmicutes species including *Streptococcus*, *Ruminococcaceae*, and *CAG-352*. The presurgery samples showed an increase in *Bacteroides*, *Bifidobacterium*, and *Agathobacter*, whereas there was again a decrease in firmicutes species including *CAG-352* and *Ruminococcaceae*. In summary, the fecal microbiota shifted during and after neoadjuvant therapy.

**Table 1.** Baseline characteristics of the included patients (n = 172) for microbiome profiling

Characteristics	No. (%)
Age, y	
Median (range)	67 (40-85)
Sex	
Male	138 (80.2)
Female	34 (19.8)
Tumor location	
Proximal or mid	22 (12.8)
Distal	124 (72.1)
Gastroesophageal junction	25 (14.5)
Not recorded	1 (0.6)
Histology	
Esophageal squamous cell carcinoma	22 (12.8)
Esophageal adenocarcinoma ^a^	150 (87.2)
Clinical tumor stage	
cT2	35 (20.3)
cT3	134 (77.9)
cT4a	1 (0.6)
cTx	2 (1.2)
Clinical nodal stage	
cN0	75 (43.6)
cN1	63 (36.6)
cN2	30 (17.4)
cN3	3 (1.7)
cNx	1 (0.6)
Enteral feeding	
Yes	8 (4.7)
No	164 (95.3)
Therapy	
Neoadjuvant chemoradiotherapy	124 (72.1)
Definitive chemoradiation^b^	8 (4.7)
Neoadjuvant chemoradiotherapy + anti-programmed cell death 1 ligand	40 (23.3)

a Two patients classified as esophageal adenocarcinoma had a mixed histology of esophageal adenocarcinoma and esophageal squamous cell carcinoma.

b Eight patients switched to definitive chemoradiation.

**Figure 3.**
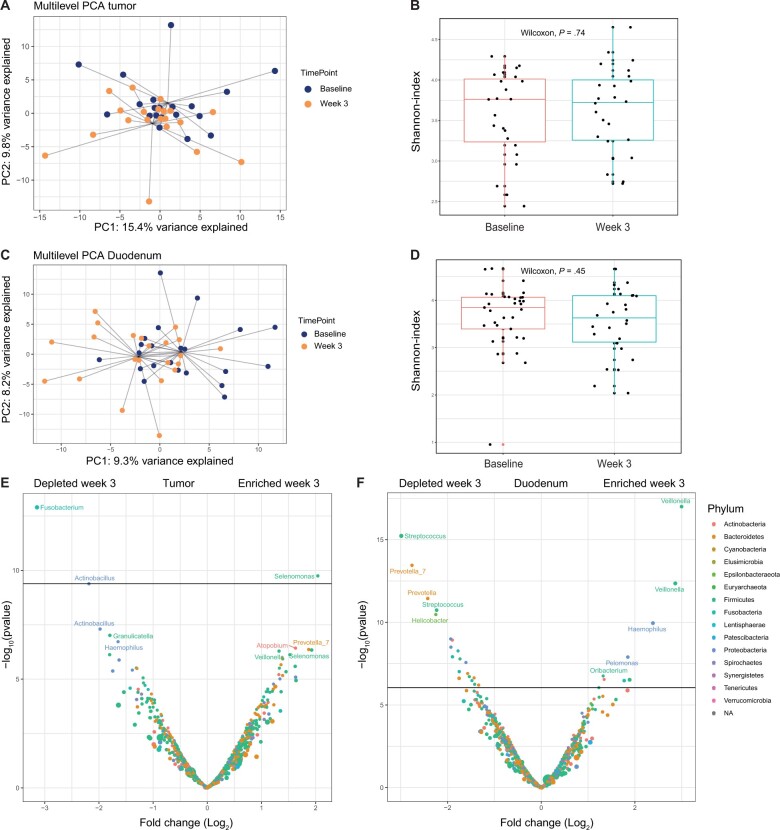
Tumor and duodenal microbiota dynamics over time in PERFECT. **A)** Multilevel principal component analysis of tumor microbiota samples over time. The colors are indicative of the 2 timepoints. **B)** Shannon index over time for the tumor microbiota samples. **C)** Multilevel principal component analysis of duodenal microbiota samples over time. The colors are indicative of the 2 timepoints. **D)** Shannon index over time for the duodenal microbiota samples. **E)** Volcano plot of tumor bacteria more or less abundant in week 3 compared with baseline. **F)** Volcano plot of duodenal bacteria more or less abundant in week 3 compared with baseline. NA = not applicable; PC1 = first principal component; PC2 = second principal component; PCA = principal components analysis.

From the PERFECT cohort, additional tumor and duodenal samples were available. The composition on genus level for the tumor and duodenal samples is given in Supplementary Figure 2, A and B (available online). First, we investigated if the tumor microbiota composition shifted during treatment. A statistically significant change in tumor microbiota profile was observed in week 3 compared with baseline (MANOVA, *P* = .014; Figure 3, A). However, the alpha diversity did not change between both timepoints (*P* = .74; Figure 3, B). Next, we investigated if the duodenal microbiota was altered over time. A statistically significant change in duodenal microbiota profile was observed in week 3 compared with baseline (MANOVA, *P* = .005; Figure 3, C). Again, alpha diversity did not change between both timepoints (*P* = .45; Figure 3, D). To compare differential abundance of individual bacteria, we compared the baseline samples with the week 3 samples. By volcano plot analysis, we observed an increase in *Selenomonas* and a decrease in *Fusobacterium* and *Actinobacillus* during treatment in the tumor samples (Figure 3, E). For the duodenal samples, there was an increase in *Veillonella*, *Haemophilus*, and *Pelomonas*, whereas there was a decrease in *Streptococcus*, *Prevotella*, and *Helicobacter* bacteria, (Figure 3, F). To conclude, the tumor and duodenal microbiota were altered in week 3 of neoadjuvant therapy.

### Microbiota composition and clinical outcomes

Having observed that the gastrointestinal microbiome changed under the influence of neoadjuvant therapy, we explored if the gut microbiota was associated with pathological complete response or PFS. To do so, we analyzed 1) fecal microbiota diversity, 2) specific fecal bacteria, and 3) duodenal and tumor microbiota composition in relation to clinical outcomes.

#### Fecal microbiome diversity

**Figure 4.**
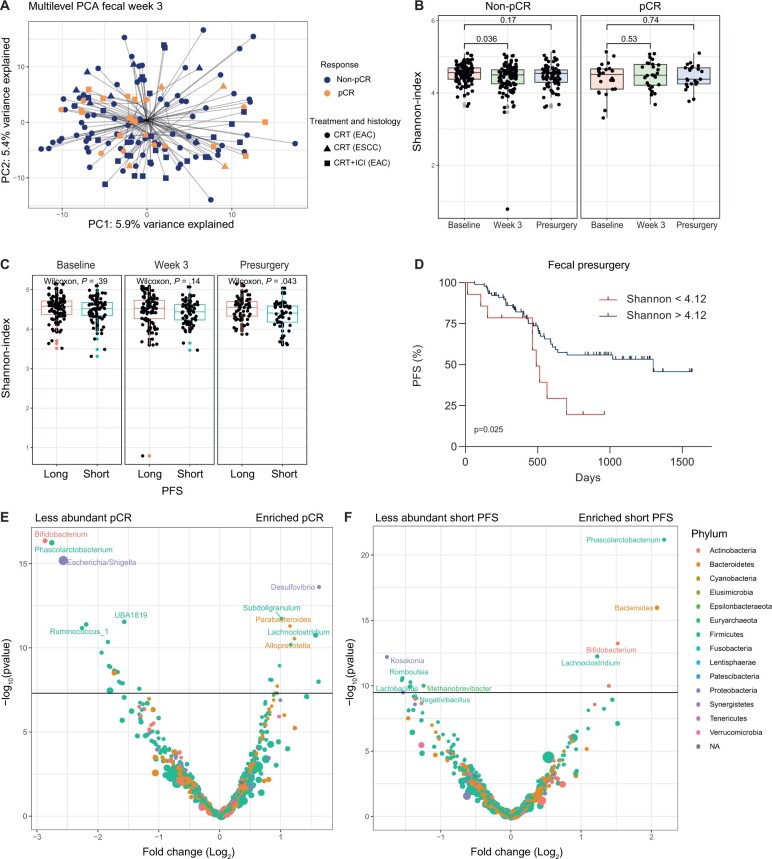
Associations between fecal microbiota and patient outcome. **A)** Multilevel principal component analysis of fecal microbiota samples in week 3. The colors are indicative of the pathological response, while symbols indicate treatment received and tumor histology. **B)** Shannon index over time for the fecal microbiota samples in patients with and without a pathological complete response. **C)** Shannon index over time for the fecal microbiota samples in patients with short and long progression-free survival (PFS). **D)** Presurgery Shannon index stratifies patients into a high- and low-risk group for PFS. A higher Shannon diversity (>4.12) was associated with a longer PFS. **E)** Week 3 volcano plot of fecal bacteria more or less abundant in patients with and without a pathological complete response. **F)** Presurgery volcano plot of fecal bacteria more or less abundant in patients with short and long PFS. CRT = chemoradiotherapy; EAC = esophageal adenocarcinoma; ESCC = esophageal squamous cell carcinoma; ICI = immune checkpoint inhibitor; NA = not applicable; PC1 = first principal component; PC2 = second principal component; PCA = principal components analysis pCR = pathological complete response.

First, we investigated if there was a baseline signature in the fecal samples associated with treatment response or PFS. There was no difference in beta (Bray–Curtis) or alpha diversity (Shannon index) that was related to pathological complete response or PFS at baseline. Also, no statistically significant difference was observed when IMPAC and PERFECT or esophageal adenocarcinoma and esophageal squamous cell carcinoma were analyzed separately. Next, we investigated if the samples during or after neoadjuvant therapy were related to clinical outcome. Interestingly, the week 3 fecal samples showed a statistically significant difference in beta diversity between patients with a pathological complete response vs the incomplete responders (*P* = .044) illustrated by PCA (Figure 4, A). Moreover, we observed a decrease in alpha diversity between baseline and week 3 in the poor response group (*P* = .036), while the pathological complete response patients were stable over time (*P* = .53) (Figure 4, B). Furthermore, we investigated if the fecal microbiota was related to PFS. There was no difference in beta diversity based on PFS for any of the timepoints, although we did observe a lower alpha diversity in patients with short PFS on the presurgery timepoint (*P* = .043) (Figure 4, C). We were able to stratify patients in a high- and low-risk group based on the Shannon value presurgery (hazard ratio [HR] = 0.44, 95% confidence interval [CI] = 0.21 to 0.92; log-rank *P* = .025) (Figure 4, D). A higher Shannon index (>4.12) was associated with longer PFS. In summary, patients with a nonpathological complete response showed 1) a statistically significant difference in beta diversity at week 3 compared with the pathological complete response group and 2) a decrease in alpha diversity after 3 weeks of neoadjuvant chemoradiotherapy. Also, a lower alpha diversity prior to surgery was associated with poor PFS.

#### Specific fecal bacteria

To investigate whether specific bacteria could differentiate between clinical outcomes, we compared specific fecal bacteria at week 3 and before surgery. In the pathological complete response group, *Desulfovibrio*, *Subdoligranulum*, and *Parabacteroides* were more abundant, whereas *Bifidobacterium*, *Phascolarctobacterium*, and *Escherichia coli* or *Shigella* were less present compared with the nonpathological complete response group (Figure 4, E). Because there was a lower Shannon index before surgery in patients with short PFS, we assessed differential abundance of bacteria on the presurgery timepoint stratified for short or long PFS. In patients with short PFS, there were more *Phascolarctobacterium*, *Bacteroides*, and *Bifidobacterium*, while there were less *Kosokonia*, *Romboutsia*, and *Lactobacillus* (Figure 4, F).

#### Tumor and duodenal microbiota

**Figure 5.**
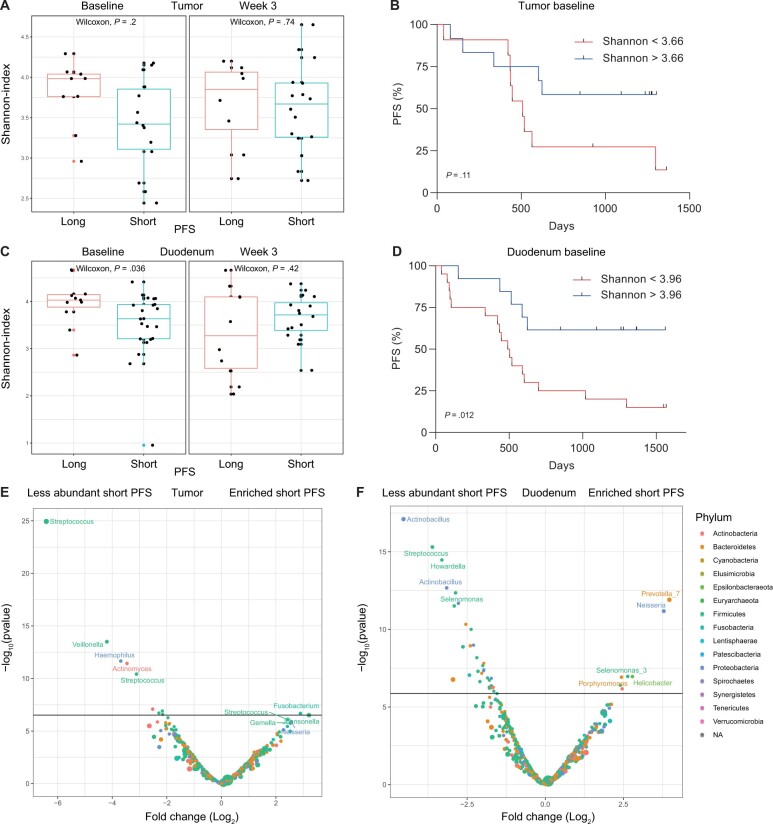
Associations between tumor, duodenal microbiota, and progression-free survival (PFS). **A)** Shannon index over time for the tumor microbiota samples in patients with short and long PFS. **B)** Baseline tumor Shannon index is not prognostic for PFS. **C)** Shannon index over time for the duodenal microbiota samples in patients with short and long PFS. **D)** Baseline duodenal Shannon index stratifies patients into a high- and low-risk group for PFS. A higher Shannon diversity (>3.96) was associated with a longer PFS. **E)** Baseline volcano plot of tumor bacteria more or less abundant in patients with short and long PFS. **F)** Baseline volcano plot of duodenal bacteria more or less abundant in patients with short and long PFS. NA = not applicable.

In the PERFECT cohort, we investigated if the tumor and duodenal microbiota were related to patient outcome. The tumor microbiota beta and alpha diversity were not statistically significantly different at baseline or week 3 for pathological complete response and PFS. There was, however, a trend towards a lower alpha diversity at baseline in patients with short PFS (*P* = .2; Figure 5, A). Baseline alpha diversity was not able to stratify patients into high- and low-risk groups (log-rank *P* = .11; Figure 5, B). Next, we investigated the duodenal microbiota and its relationship with patient outcome. There was no difference in beta diversity for pathological complete response and PFS for both timepoints. However, there was a difference in duodenal alpha diversity at baseline, which was lower in patients with short PFS (*P* = .036; Figure 5, C). In addition, the baseline duodenal alpha diversity was able to stratify patients into high- and low-risk groups based on Kaplan–Meier analysis (HR = 0.30, 95% CI = 0.11 to 0.81; log-rank *P* = .012; Figure 5, D). A higher Shannon index (>3.96) was associated with longer PFS.

Additionally, to investigate whether individual tumor bacteria were related to PFS we assessed the differential abundance of tumor bacteria and at baseline found more *Fusobacterium* in patients with short PFS and less *Streptococcus*, (Figure 5, E). Interestingly, *F nucleatum* previously associated with chemotherapy resistance in esophageal squamous cell carcinoma was also more abundant in patients with short PFS, although not statistically significant (*P* *= .*07) (23). To investigate whether bacteria were over- or underrepresented in baseline duodenal biopsies, we also assessed differential abundance according to PFS status. Patients with a short PFS had, among others, more *Prevotella*, *Neisseria*, and *Helicobacter* bacteria and less *Actinobacillus*, *Streptococcus*, and *Howardella*, (Figure 5, F). In conclusion, we identified specific tumor bacteria at baseline associated with poor clinical outcome (PFS) and found that lower duodenal alpha diversity was associated with early progression or death.

## Discussion

This is the first prospective study that analyzed the relationship between microbiome composition (fecal, tumor, and duodenal) and treatment outcomes in patients with resectable esophageal carcinoma. In line with previous studies, we observed a shift in gastrointestinal microbiome composition in all patients after neoadjuvant therapy. However, a more stable fecal alpha diversity was associated with a better response and PFS (compared with patients with a decrease in alpha diversity). Specific for patients treated with a combination of neoadjuvant chemoradiotherapy and checkpoint blockade, lower baseline duodenal alpha diversity was related to early progression. Additionally, we identified specific fecal, duodenal, and tumor bacteria associated with these clinical outcomes. These results emphasize the importance of microbiome composition and how it can affect treatment effectivity.

The fecal, tumor, and duodenal microbiota shifted over time in esophageal carcinoma patients, most likely under the influence of neoadjuvant therapy. Chemotherapy can cause gut dysbiosis and thereby have a profound influence on different functional metabolism-related pathways (5,37,38). Illustrative is a randomized study with 61 esophageal carcinoma patients treated with neoadjuvant docetaxel, cisplatin, and 5-fluorouracil investigating the effect of synbiotics (pre- and probiotics) on gut microbiota composition (38). The control group experienced a decrease in several beneficial strains and an increase in harmful *Clostridium difficile* and *Enterococcus* bacteria (38). Interestingly, the synbiotic group had a more favorable ratio of beneficial vs harmful bacteria. In our study, patients received not only chemotherapy but also radiation and, additionally, atezolizumab in the PERFECT cohort. Chemoradiotherapy has also been implicated as a gut microbiome modulator in lung cancer and rectal cancer (17,39). Reductions in alpha diversity were observed over time in both tumor types. Also, checkpoint inhibition can lead to fluctuations in gut microbiome composition related to therapy response (40). In addition to the fecal microbiota, we also observed changes in the tumor and duodenal bacterial composition in the third week of therapy. This has also been observed in anal cancer patients treated with chemoradiation with enrichment of *Clostridia* and *Corynebacterum* in anorectal swabs of the tumor site during and after treatment (18). In summary, the intestinal and tumor microbiota of esophageal carcinoma patients were altered by neoadjuvant therapy.

Interestingly, the week 3 gut microbiota of poor responders was different from complete responders and decreased in alpha diversity compared with baseline. There were more *E coli* and *Shigella* bacteria in poor responders. Whether these findings have a mechanistic causal relationship or are related to general health status could not be established because of the observational nature of our study. However, findings from previous studies seem to support a causal relationship between the gut microbiota and chemoradiotherapy response (6,15,17,39,41). In rectal cancer patients treated with neoadjuvant chemoradiotherapy *Bacteroides vulgatus*–mediated nucleotide biosynthesis conferred resistance to 5-fluorouracil and irradiation (39). This was based on gut microbiota sequencing of 353 samples and in vitro and in vivo functional validation. In lung cancer patients treated with docetaxel and nedaplatin-based chemoradiotherapy, the fecal Shannon alpha diversity index was lower during treatment in patients who had a PFS of less than 11 months (17). This is in line with our findings where a reduction in Shannon was observed in patients with a poor response (17). A lower Shannon index has previously been associated with poor health status including obesity, inflammation (C-reactive protein, interleukin-6), sugary drinks, and diarrhea (42). Future studies should explore if interventions aimed at enhancing gut microbiome diversity can improve neoadjuvant chemoradiotherapy response. To achieve this, mechanistic in vivo studies are needed as well as patient intervention studies with, for example, freeze-dried oral microbiota capsules from healthy donors or responders (43-45). These capsules may offer a more patient-friendly and repeatable alternative compared with conventional fecal microbiota transplantation.

In the PERFECT cohort, we observed a correlation between baseline tumor and duodenal microbiota composition and PFS although this was not observed for the baseline fecal microbiota. This suggests microbes in the vicinity of the tumor could be more important determinants of outcome in esophageal carcinoma patients treated with neoadjuvant checkpoint blockade. In the tumor biopsies, we observed an overrepresentation of *Fusobacterium* in patients with short PFS. This bacterium plays an important role in colorectal cancer growth and was detected in primary and metastatic lesions (46). In pancreatic cancer, the intratumoral presence of *F nucleatum* was associated with CD8^+^ T-cell suppression by recruiting myeloid-derived suppressor cells to the tumor microenvironment in a subcutaneous mouse model (47). In esophageal squamous cell carcinoma, *F nucleatum* was responsible for tumor progression by nuclear factor-κB activation (48). A recent pan-cancer analysis of the tumor microbiome derived from genomic data found *Fusobacterium* to be associated with immune checkpoint inhibitor resistance in non–small cell lung carcinoma (49). Moreover, tumors with a high abundance of *Fusobacterium* had lower gene expression signatures of cytotoxic T lymphocytes, interferon-y, and major histocompatibility complex class II (49). These findings seem to suggest *Fusobacterium* is contributing to shaping an immunosuppressive tumor microenvironment. How *Fusobacterium* is related to esophageal adenocarcinoma progression is unknown but could be through inhibition of cytotoxic T cells and the recruitment of immunosuppressive immune cells. Further research is needed to elucidate how *Fusobacterium* is related to esophageal adenocarcinoma progression. Additionally, lower alpha diversity of duodenal samples and presence of *Prevotella* and *Neisseria* were related to poor PFS. Previously, decreasing duodenal microbiota diversity has been associated with aging, chronic inflammation, and concomitant diseases (50). Duodenal microbiota diversity may thus also be a prognostic marker for esophageal adenocarcinoma cancer progression. Whether these findings have a causal relationship with tumor progression or are general indicators of health status should be further investigated. A potential lead for further study is the role of resident memory CD8^+^ T cells in the duodenum and if these are affected by the local microbiota (51). These T cells can induce local tumor immunity and have been associated with response to immunotherapy (52). In conclusion, the microbiota of tumor and duodenum are associated with PFS and require further mechanistic validation to develop clinical interventions beneficial for esophageal adenocarcinoma patients.

Several limitations of this study must be considered. First, we collected fecal samples from esophageal squamous cell carcinoma and esophageal adenocarcinoma patients. In general, esophageal squamous cell carcinoma is more sensitive to neoadjuvant chemoradiotherapy, and therefore a different relationship might exist between the fecal microbiota and tumor response (2). This study was not powered to investigate if this was the case with only a limited number of esophageal squamous cell carcinoma patients. However, based on the composition of the fecal microbiota assessed by PCA, we found no difference between esophageal squamous cell carcinoma and esophageal adenocarcinoma patients. Second, our study might be underpowered to detect subtle differences between bacterial species. We only included a limited number of tumor and duodenal samples. Third, our results were not corrected for confounders known to affect the microbiome including smoking, diabetes mellitus, and antibiotics used during treatment (53). Fourth, we were not able to correlate our results with overall survival because of limited follow-up in several patients. Although, PFS is usually well correlated with long-term survival in this patient group, a future study should perform additional correlative analyses for overall survival (54).

Fecal, tumor, and duodenal microbiota of patients with resectable esophageal carcinoma shifted during and after neoadjuvant therapy. Strikingly, our study shows that a more stable microbial diversity despite neoadjuvant treatment is associated with a better response and PFS. Furthermore, baseline tumor and duodenal microbiota composition were related to PFS in patients treated with neoadjuvant chemoradiotherapy and atezolizumab. Future intervention studies that alter, improve or modulate the intestinal and tumor microbiome are needed to validate these results and ultimately improve treatment response.

## Supplementary Material

djae153_Supplementary_Data

## Data Availability

The data underlying this article can be found in the European Nucleotide Archive (PRJEB76265).
