# Supplementary material for: Fecal, duodenal, and tumor microbiota composition of esophageal carcinoma patients, a longitudinal prospective cohort
Source: J Natl Cancer Inst. 2024 Jun 26;116(11):1834–44. doi: 10.1093/jnci/djae153 (PMC11542985; doi:10.1093/jnci/djae153)
Supplement: djae153_Supplementary_Data [file djae153_supplementary_data.zip › djae153_Supplementary_Data/Supplementary Figure 2..pdf]

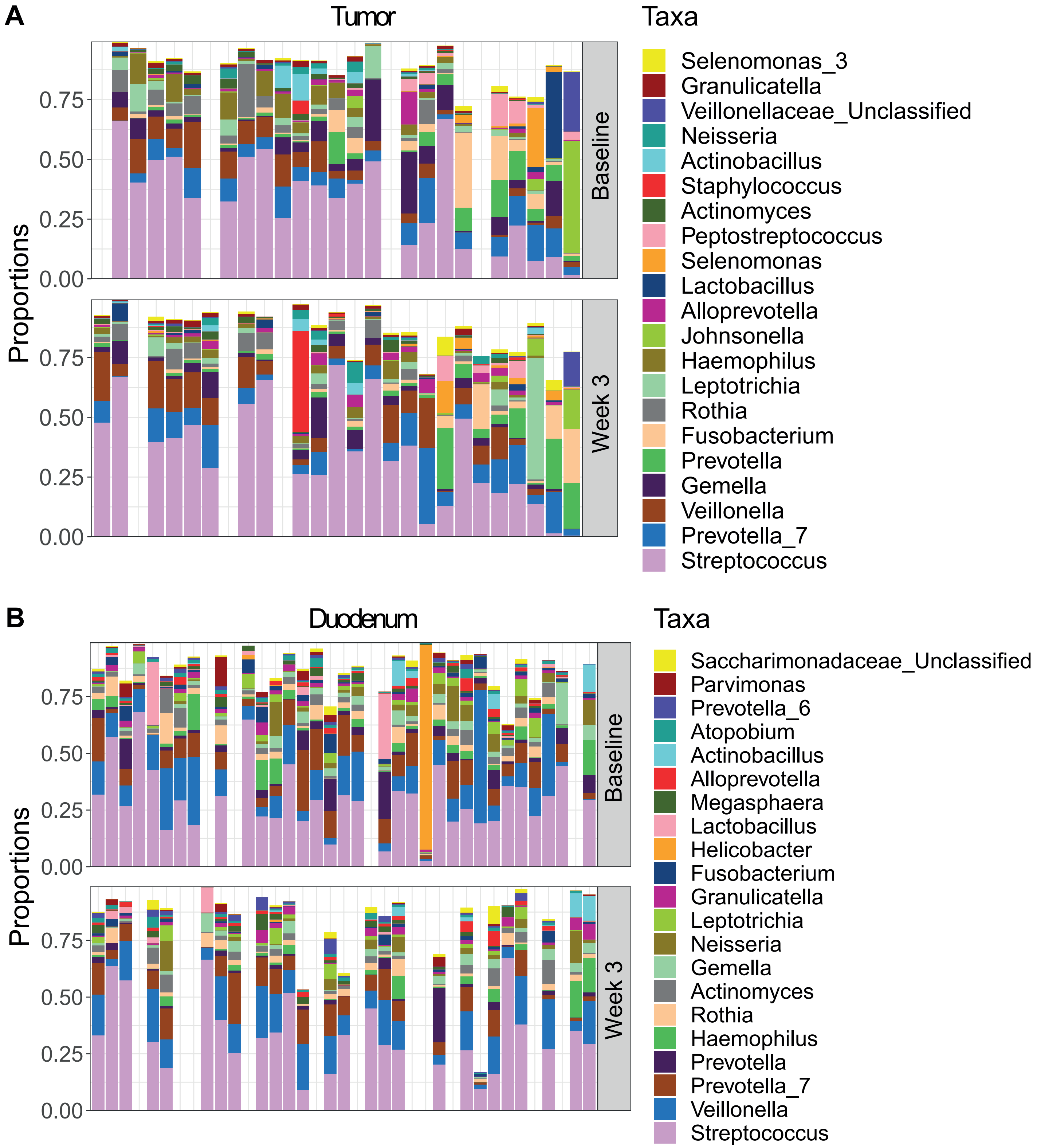
A
Tumor
Taxa
Selenomonas_3
Granulicatella
Veillonellaceae_Unclassified
Neisseria
Actinobacillus
Staphylococcus
Actinomyces
Peptostreptococcus
Selenomonas
Lactobacillus
Alloprevotella
Johnsonella
Haemophilus
Leptotrichia
Rothia
Fusobacterium
Prevotella
Gemella
Veillonella
Prevotella_7
Streptococcus
Proportions
0.00
0.25
0.50
0.75
Baseline
Week 3
B
Duodenum
Taxa
Saccharimonadaceae_Unclassified
Parvimonas
Prevotella_6
Atopobium
Actinobacillus
Alloprevotella
Megasphaera
Lactobacillus
Helicobacter
Fusobacterium
Granulicatella
Leptotrichia
Neisseria
Gemella
Actinomyces
Rothia
Haemophilus
Prevotella
Prevotella_7
Veillonella
Streptococcus
Proportions
Baseline
Week 3
