# Supplementary figures and images for: Fecal, duodenal, and tumor microbiota composition of esophageal carcinoma patients, a longitudinal prospective cohort
Source: J Natl Cancer Inst. 2024 Jun 26;116(11):1834–44. doi: 10.1093/jnci/djae153 (PMC11542985; doi:10.1093/jnci/djae153)

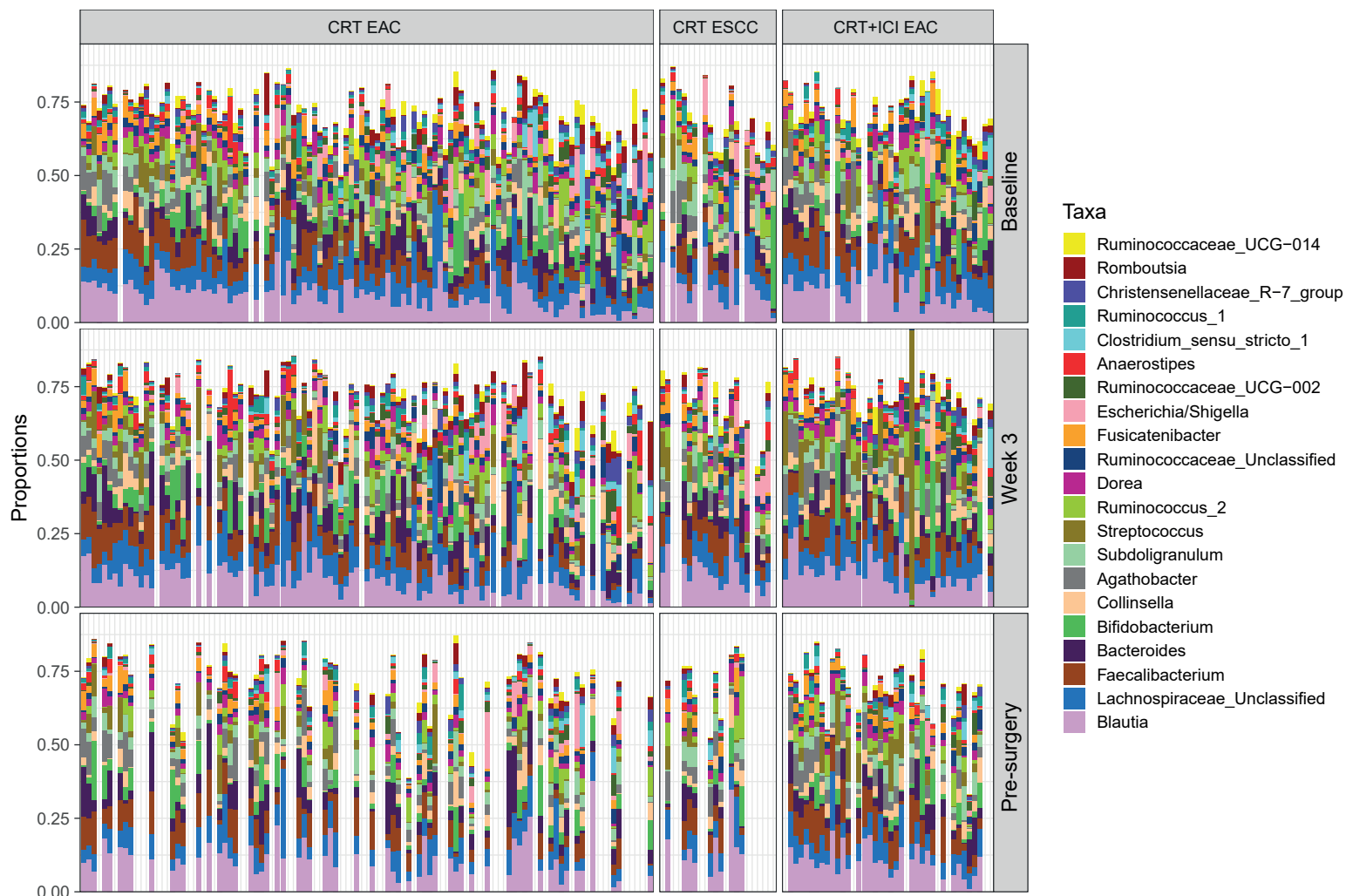

Supplement: djae153_Supplementary_Data [file djae153_supplementary_data.zip › djae153_Supplementary_Data/Supplementary Figure 1..pdf]
